# Genetic variation of *E6* and *E7* genes of human papillomavirus type 16 from central China

**DOI:** 10.1186/s12985-023-02188-8

**Published:** 2023-09-27

**Authors:** Ting Li, Zhiping Yang, Chunlin Zhang, Sutong Wang, Bing Mei

**Affiliations:** Department of Laboratory Medicine, Jingzhou Hospital Affiliated to Yangtze University, Jingzhou, 434020 China

**Keywords:** Human papillomavirus type 16 (HPV-16), Sequencing, Variation, Epitope prediction, Selection pressure

## Abstract

**Background:**

Persistent high-risk human papillomavirus (HR-HPV) infection is an important factor in the development of cervical cancer, and human papillomavirus type 16 (HPV-16) is the most common HR-HPV type worldwide. The oncogenic potential of HPV-16 is closely related to viral sequence variation.

**Methods:**

In order to clarify the variant characteristics of HPV-16 *E6* and *E7* genes in central China, *E6* and *E7* sequences of 205 HPV‐16 positive samples were amplified by polymerase chain reaction. PCR products of *E6* and *E7* genes were further sequenced and subjected to variation analysis, phylogenetic analysis, selective pressure analysis and B-cell epitope prediction.

**Results:**

Twenty-six single nucleotide variants were observed in *E6* sequence, including 21 non-synonymous and 5 synonymous variants. Twelve single nucleotide variants were identified in *E7* sequence, including 6 non-synonymous and 6 synonymous variants. Four new variants were found. Furthermore, nucleotide variation A647G (N29S) in *E7* was significantly related to the higher risk of HSIL and cervical cancer. Phylogenetic analysis showed that the *E6* and *E7* sequences were all distributed in A lineage. No positively selected site was found in HPV-16 *E6* and *E7* sequences. Non-conservative substitutions in *E6*, H31Y, D32N, D32E, I34M, L35V, E36Q, L45P, N65S and K75T, affected multiple B-cell epitopes. However, the variation of *E7* gene had little impact on the corresponding B-cell epitopes (score < 0.85).

**Conclusion:**

HPV-16 *E6* and *E7* sequences variation data may contribute to HR-HPV prevention and vaccine development in Jingzhou, central China.

## Background

Cervical cancer is one of the most frequently cancer in women worldwide, with an estimated 604,000 new cases and 342,000 deaths worldwide in 2020 [1]. High-risk human papillomavirus (HR-HPV) infection is the main cause of cervical cancer, which including 15 common types (16, 18, 52, 58, 68, 66, 56, 31, 33, 35, 45, 82, 39, 51,59) [2]. The pathogenicity of HPV types is associated with a variety of factors including geography and ethnicity. Among these HR-HPV types, HPV-16 is the one with the highest carcinogenic risk in cervical cancer cases [3].

HPV is a double-stranded circular DNA virus with a genome of roughly 8000 base pairs, having three functional regions: (1) long control region (LCR); (2) early gene region (*E*): *E1*, *E2*, *E4*–*E7*, which encode *E1*, *E2*, *E4*–*E7* early proteins, respectively; (3) late gene region (*L*): composed of the ORFs of *L1* and *L2*, encoding *L1* and *L2* late proteins [4]. The viral *E6* and *E7* oncogenes play central roles in both HPV-induced oncogenesis initiation and malignant growth of HPV-positive cancer cells through the encoded E6 and E7 protein, which bind to p53 tumor suppressor protein and retinoblastoma gene product (pRb) respectively and result in degradation of the suppressor protein and the following cell proliferation and tumor formation [5].

Sequence diversity exists in each HPV type, with intra-type variation spectra varying from 1 to 10% and sub-spectra varying from 0.5 to 1% [6]. There are 4 main lineages (A, B, C, D) and 16 sub-lineages of HPV-16, including A1–3 (European), A4 (Asian), B1–4 (African 1), C1–4 (African 2), D1 (North American), D2–3 (Asian-American) and D4 [7]. HPV-16 intra-type variants are preferentially associated with specific histological types of cancer, and non-European HPV-16 variants have a higher oncogenicity due to their association with high-grade lesions of the cervix and invasive tumors [8, 9]. In one study, the association of HPV variant lineages with precancer and cancer is analyzed, showing that the presence of HPV-16 A4 variants is significatively associated with an increased risk of cervical cancer, compared to A1/A2; while women with non-A sub-lineages (B, C, D) have a higher risk of CIN 3 and cervical cancer [10]. Furthermore, molecular and epidemiological data indicate that variants of the same HPV type are biologically distinct and may confer differential pathogenic risks. HPV-16 *E6* variant E-G350 is closely related to HPV-16 persistent infection and cervical lesions transformation from LSIL to HSIL [11]. High mutation rates of HPV-16 *E6* L83V are associated with viral persistence and cervical intraepithelial neoplasia progression, conferring a higher risk for high-grade cervical intraepithelial neoplasia development [11]. The HPV-16 *E7* variant A647G (N29S) has been observed previously in some cervical cancer cases, and shows an increased oncogenicity in vitro, though at present cervical cancer risk association is conflicting across the various studies [12–14].

HPV prevalence and type distribution vary across the countries and the different regions of a country [15]. HPV-16 is the most oncogenic HPV type, with relevance to above 50% of cervical cancer worldwide [16]. The aims of this study were to identify gene variations and corresponding amino acid variations in HPV-16 *E6* and *E7* gene in Jingzhou, whose longitude and latitude are 111° 15′–114° 05′ E and 29° 26′–31° 37′ N respectively, west of Wuhan city in central China, construct phylogenetic trees of *E6* and *E7* sequences, predict the secondary structure and B-cell epitopes of the proteins, and perform selective pressure analysis. The results of this study may provide useful data for the prevention and treatment of HPV in Jingzhou area, central China.

## Methods

### Sample collection

In this study, cervical exfoliated cell samples were collected from the female patients underwent cervical screening of possible HR-HPV infection at gynecology department of Jingzhou Hospital Affiliated to Yangtze University from September 2019 to October 2021. Informed consents were obtained from all patients and the study was approved by the Ethics Committee of Jingzhou Hospital Affiliated to Yangtze University.

### DNA extraction and typing

Based on the method of magnetic beads, DNA was extracted according to the instruction of the nucleic acid extracting kit (Guangzhou Magen Biotechnology Co., Ltd.). The DNA extraction products were measured by real-time quantitative PCR according to the instructions of HR-HPV typing kit (Shanghai ZJ Bio-Tech Co., Ltd.), covering 15 HR-HPV types (16, 18, 52, 58, 68, 66, 56, 31, 33, 35, 45, 82, 39, 51, 59). 4μL extraction products, as PCR template, were used for HPV typing. Amplification parameters were initially 94 °C for 2 min, 93 °C for 10 s, and 62 °C for 31 s for 40 cycles. In each DNA amplification reaction, the total amount of product after each PCR cycle was measured with a fluorescent chemical at 62 °C. Both positive and negative controls were used for PCR amplification. Finally, the remained extracted DNA products were stored in − 80 °C for subsequent experiments.

### PCR amplification and sequencing

In this study, samples with single positive HPV-16 were selected to further analyze the variation of HPV-16 sequence. The HPV-16 *E6* and *E7* sequence-specific primers were designed using Primer Premier 6.0 according to the reference sequence of the HPV-16 prototype (GenBank: K02718). The primers were synthesized by Sangon Biotech (Shanghai). The PCR amplification system was ddH_2_O 15.875 μL, 10 × Buffer (MgCl_2_) (Takara) 2.5 μL, dNTP (Takara) 2 μL, Taq polymerase (5 U/L) (Takara) 0.125 μL, forward primer (20 μM) 1.25 μL, reverse primer (20 μM) 1.25 μL and DNA extraction 2.0 μL. PCR amplification parameters included pre-denaturation 95 °C for 3 min, denaturation at 94 °C for 45 s, annealing at 59.3 °C (*E6*)/58.9 °C (*E7*) for 45 s, extension at 72 °C for 60 s for 35 cycles, and final extension at 72 °C for 10 min. PCR products were subjected to 2.5% agarose gel electrophoresis, stained with gel red nucleic acid dye (Biotium) and sent to Sangon Biotech for sequencing. The following primers were used:

- HPV-16 E6-F: 5′-CTAAGGGCGTAACCGAAATCG-3′;
- HPV-16 E6-R: 5′-TGCTCATAACAGTAGAGATCAGTTG-3′;
- HPV-16 E7-F: 5′-CCACTGTGTCCTGAAGAA-3′;
- HPV-16 E7-R: 5′-TCACCTGTATCACTGTCATT-3′.

#### Variation analysis

The sequencing results were examined for peak plots using Chromas software. Variation analysis of *E6* and *E7* variants was performed in comparison with the reference sequence of HPV-16 on NCBI (GenBank: K02718) through MEGA11 software. The variant sequences were further transformed into amino acid sequences to analyze the variation of the encoded proteins and predict their secondary structures using GOR4.

### Phylogenetic analysis

The Maximum Likelihood tree was constructed using MEGA11 software with repeat parameters set to 1000 times. The reference sequences of HPV-16 lineages downloaded from NCBI were used to construct phylogenetic branches, including K02718 (A1), HQ644284 (A1), AF536179 (A2), HQ644236 (A3), AF534061 (A4), HQ644235 (A4), KU053908 (B1), HQ644238 (B1), HQ644298 (B2), KU015 (B3), KU053914 (B4), KU053917 (C1), HQ644239 (C1), HQ644244 (C2), KU053920 (C3), KU053925 (C4), HQ644257 (D1), AY686579 (D2), HQ644279 (D2), AF402678 (D3), HQ644269 (D3) and KU053931 (D4) [17].

### Selective pressure analysis

The codeML program in pamlX software based on Maximum likelihood method (http://abacus.gene.ucl.ac.uk/software/paml.html) was used to infer dn/ds and positively selected site. Bayesian empirical Bayesian method was also used to calculate the posterior probability of positively selected site. Seven codon-substitution models (M0, M1, M2, M3, M5, M7, M8) were used to determine whether positive selection impacts the evolution of *E6–E7*. These models treat codons as basic units of evolutionary change and take into account genealogical history when calculating parameters. The log-likelihood score evaluates the quality of fit of the input data to the model conditions. These seven codon models use different assumptions to estimate different sets of parameters. In these models, dn/ds, estimated as separate cryptographic subclasses, are assumed to evolve independently of each other [18]. To assess the effect of the positive selection on a specific coding region, a likelihood ratio test (LRT) was performed to compare the nested models [19].

### B-cell epitope prediction

B-cell epitopes of HPV-16 E6 and E7 reference and variant sequences were predicted using ABCpred Server (http://crdd.osdd.net/raghava/abcpred/), an artificial neural network-based B-cell epitope prediction server, based on default parameters. The higher the prediction score, the better the affinity of the epitope.

## Results

### Epidemiological characteristics of HR-HPV in Jingzhou

Epidemiological and typing data of HR-HPV were investigated in samples collected from January 2018 to December 2021 in Jingzhou, central China. Out of 31,633 samples tested, 6014 were positive for HR-HPV (19.01%). HPV-52 was the most common HR-HPV type (2029, 6.41%), followed by HPV-58 (1048, 3.31%) and HPV-16 (843, 2.66%).

### HPV-16 *E6* and *E7* variation

Two hundred and five HPV-16 single positive samples were amplified and sequenced, of which *E6* and *E7* sequences were successfully obtained for 176 samples (median age 43.5 years; range 18–68 years), and the other 29 samples were excluded due to PCR or sequencing failure. In 176 samples,110 women were histological normal (62.5%), 9 were LSIL (5.1%), 42 were HSIL (23.9%) and 15 were cervical cancer (8.5%). Among the 176 *E6* and *E7* sequences, 168 sequences had nucleotide variants, and the remaining 8 sequences were completely homologous to the reference sequence. In this study, the *E6* and *E7* variant sequences were further divided into 45 different variant groups, noted as 16HB01–16HB45, and the same sequences represented a specific variant group. These sequence data were submitted to GenBank with accession numbers OQ659416–OQ659460. 16HB01 (58/168, 34.5%) and 16HB02 (24/168, 14.3%) were the sequences with the highest variation frequency. Thirty-eight single nucleotide variants were identified in the *E6* and *E7* sequence, including 27 nonsynonymous variants and 11 synonymous variants. The sequence variability of *E6* gene was higher than that of *E7* gene. The most common nucleotide variation in *E6* was T178G (D32E) (102/168, 60.7%), while the most common nucleotide variations in *E7* were A647G (N29S) (101/168, 60.1%) and T846C (102/168, 60.7%). Distribution of major nucleotide variation in HPV16 E6 and E7 based on cervical disease status was shown in Table 1. We combined the groups of histological normal and LSIL into one category, meanwhile, combined HSIL and cervical cancer into another category. The statistical results suggested that A647G (N29S) was significantly related to HSIL and cervical cancer (*OR* = 1.99*, *95% CI = 1.03 to 3.87*, P* = 0.04). T292C of *E6* sequence and G597A, G753C and G781C of *E7* sequence were novel variants. Five amino acid variants, including two non-conservative substitutions, were found in the sequence affecting the alpha helix. Ten amino acid variants, all with non-conservative substitutions, were found in the sequence affecting the fold. The results were shown in Table 2.

**Table 1:** Distribution of major nucleotide variation in HPV16 E6 and E7 genes based on cervical disease status

Gene	Nucleotide variation	Women with cervical disease status				
		HN	LSIL	HSIL	CC	Total
		n = 110	n = 9	n = 42	n = 15	n = 176
E6	G94A	8	1	2	0	11
	A95G	0	0	1	0	1
	T109C	1	0	0	0	1
	A131C	2	0	0	0	2
	T137G	1	0	0	0	1
	C153T	0	0	0	1	1
	C158A	0	0	1	0	1
	C173T	1	0	0	0	1
	G176A	1	1	0	0	2
	T178G	60	3	30	9	102
	T178A	3	0	1	0	4
	A184G	0	0	1	0	1
	T185G	5	0	1	2	8
	G188C	1	1	1	0	3
	T216C	1	0	0	0	1
	T241G	2	0	1	0	3
	A276G	2	0	1	2	5
	T292C	1	0	0	0	1
	A306C	1	0	0	0	1
	C335T	2	0	2	0	4
	T350G	8	0	1	1	10
	C387T	1	0	0	0	1
	G395T	0	0	0	1	1
	A426C	0	0	0	1	1
	A442C	7	2	4	0	13
	G489A	0	0	0	1	1
	G553T	0	0	1	0	1
E7	G597A	0	0	1	0	1
	A646C	6	1	0	1	8
	A647G	59	3	30	9	101
	G663A	2	0	3	0	5
	G666A	13	1	4	5	23
	G753C	1	1	0	0	2
	T760C	10	2	1	0	13
	G781C	0	1	0	0	1
	C790T	4	0	4	0	8
	G823C	1	0	0	0	1
	T843C	15	1	6	4	26
	T846C	60	3	30	9	102
	Total	279	21	127	46	473

HN, histological normal; LSIL, low-grade squamous intraepithelial lesion; HSIL, high-grade squamous intraepithelial lesion; CC, cervical cancer

**Table 2:** The variations of HPV16 *E6* and *E7* genes

Positions	*E6*																										*E7*												N (176)
	94	95	109	131	137	153	158	173	176	178	184	185	188	216	241	276	292	306	335	350	387	395	426	442	489	553	597	646	647	663	666	753	760	781	790	823	843	846	
Reference	G	A	T	A	T	C	C	C	G	T	A	T	G	T	T	A	T	A	C	T	C	G	A	A	G	G	G	A	A	G	G	G	T	G	C	G	T	T	8
16HB01	–	–	–	–	–	–	–	–	–	G	–	–	–	–	–	–	–	–	–	–	–	–	–	–	–	–	–	–	G	–	–	–	–	–	–	–	–	C	58
16HB02	–	–	–	–	–	–	–	–	–	G	–	–	–	–	–	–	–	–	–	–	–	–	–	–	–	–	–	–	G	–	–	–	–	–	–	–	C	C	24
16HB03	–	–	–	–	–	–	–	–	–	–	–	–	–	–	–	–	–	–	–	–	–	–	–	–	–	–	–	–	–	–	–	–	C	–	–	–	–	–	10
16HB04	–	–	–	–	–	–	–	–	–	–	–	–	–	–	–	–	–	–	–	–	–	–	–	–	–	–	–	–	–	–	–	–	–	–	T	–	–	–	6
16HB05	–	–	–	–	–	–	–	–	–	–	–	–	–	–	–	–	–	–	–	–	–	–	–	–	–	–	–	–	–	–	A	–	–	–	–	–	–	–	6
16HB06	–	–	–	–	–	–	–	–	–	–	–	–	–	–	–	G	–	–	–	–	–	–	–	–	–	–	–	–	–	–	A	–	–	–	–	–	–	–	5
16HB07	–	–	–	–	–	–	–	–	–	G	–	–	–	–	–	–	–	–	–	–	–	–	–	C	–	–	–	–	G	A	–	–	–	–	–	–	–	C	5
16HB08	–	–	–	–	–	–	–	–	–	G	–	G	–	–	–	–	–	–	–	–	–	–	–	–	–	–	–	–	G	–	–	–	–	–	–	–	–	C	5
16HB09	–	–	–	–	–	–	–	–	–	–	–	–	–	–	–	–	–	–	–	G	–	–	–	–	–	–	–	–	–	–	–	–	–	–	–	–	–	–	3
16HB10	–	–	–	–	–	–	–	–	–	–	–	–	–	–	G	–	–	–	–	–	–	–	–	–	–	–	–	–	–	–	A	–	–	–	–	–	–	–	3
16HB11	–	–	–	C	–	–	–	–	–	G	–	–	–	–	–	–	–	–	–	–	–	–	–	–	–	–	–	–	G	–	–	–	–	–	–	–	–	C	2
16HB12	A	–	–	–	–	–	–	–	–	A	–	–	–	–	–	–	–	–	–	–	–	–	–	–	–	–	–	–	–	–	A	–	–	–	–	–	–	–	2
16HB13	–	–	–	–	–	–	–	–	–	–	–	–	–	–	–	–	–	–	–	–	–	–	–	C	–	–	–	–	C	–	–	–	–	–	–	–	–	–	2
16HB14	–	–	–	–	–	–	–	–	–	–	–	–	C	–	–	–	–	–	–	–	–	–	–	–	–	–	–	–	–	–	–	–	–	–	–	–	–	–	2
16HB15	–	–	–	–	–	–	–	–	–	–	–	G	–	–	–	–	–	–	–	–	–	–	–	–	–	–	–	–	–	–	A	–	–	–	–	–	–	–	2
16HB16	A	–	–	–	–	–	–	–	–	–	–	–	–	–	–	–	–	–	–	G	–	–	–	–	–	–	–	–	–	–	–	–	–	–	–	–	–	–	2
16HB17	–	–	–	–	–	–	–	–	–	–	–	–	–	–	–	–	–	–	T	–	–	–	–	C	–	–	–	C	–	–	–	–	–	–	–	–	–	–	2
16HB18	A	–	–	–	–	–	–	–	–	–	–	–	–	–	–	–	–	–	–	–	–	–	–	–	–	–	–	–	–	–	–	–	–	–	–	–	–	–	2
16HB19	–	–	–	–	–	–	–	T	–	–	–	–	–	–	–	–	–	–	–	–	–	–	–	–	–	–	–	–	–	–	–	–	C	–	–	–	–	–	1
16HB20	–	–	–	–	G	–	–	–	–	G	–	–	–	–	–	–	–	–	–	–	–	–	–	–	–	–	–	–	G	–	–	–	–	–	–	–	–	C	1
16HB21	–	–	–	–	–	–	–	–	–	–	–	–	–	–	–	–	–	–	–	–	–	–	–	C	–	–	–	–	–	–	A	–	–	–	–	–	–	–	1
16HB22	–	–	–	–	–	–	–	–	–	–	–	–	–	–	–	–	C	–	–	–	–	–	–	C	–	–	–	C	–	–	–	–	–	–	–	–	–	–	1
16HB23	A	–	–	–	–	–	–	–	–	A	–	–	–	–	–	–	–	C	–	–	–	–	–	–	–	–	–	–	–	–	A	–	–	–	–	–	–	–	1
16HB24	A	–	–	–	–	–	–	–	–	–	–	–	–	–	–	–	–	–	–	–	–	–	–	–	–	–	A	–	–	–	–	–	–	–	T	–	–	–	1
16HB25	–	–	–	–	–	–	–	–	–	G	–	–	–	–	–	–	–	–	–	–	–	–	–	–	–	–	–	–	–	–	–	–	–	–	–	–	–	C	1
16HB26	–	–	–	–	–	–	–	–	–	–	–	–	C	–	–	–	–	–	–	–	–	–	–	C	–	–	–	C	–	–	–	–	–	–	–	–	–	–	1
16HB27	–	–	–	–	–	–	–	–	–	G	–	–	–	–	–	–	–	–	–	–	–	T	–	–	–	–	–	–	G	–	–	–	–	–	–	–	–	C	1
16HB28	–	–	–	–	–	–	–	–	–	–	–	–	–	–	–	–	–	–	–	–	T	–	–	–	–	–	–	–	–	–	–	–	–	–	–	–	–	–	1
16HB29	–	–	–	–	–	–	–	–	–	–	–	–	–	–	–	–	–	–	–	G	–	–	–	–	A	–	–	C	–	–	–	–	–	–	–	–	–	–	1
16HB30	–	–	–	–	–	–	–	–	–	–	–	–	–	–	–	–	–	–	–	–	–	–	C	–	–	–	–	–	–	–	A	–	–	–	–	–	–	–	1
16HB31	–	–	–	–	–	–	–	–	–	–	–	–	–	–	–	–	–	–	–	G	–	–	–	–	–	–	–	–	–	–	–	C	–	–	–	–	–	–	1
16HB32	–	–	–	–	–	–	–	–	A	–	–	–	–	–	–	–	–	–	–	–	–	–	–	–	–	–	–	–	–	–	–	C	C	C	–	–	–	–	1
16HB33	A	–	–	–	–	–	–	–	–	G	–	–	–	–	–	–	–	–	–	–	–	–	–	–	–	–	–	–	–	–	A	–	–	–	–	–	–	–	1
16HB34	–	–	–	–	–	–	A	–	–	G	–	G	–	–	–	–	–	–	–	–	–	–	–	–	–	–	–	–	G	–	–	–	–	–	–	–	–	C	1
16HB35	–	–	–	–	–	–	–	–	–	–	–	–	–	–	–	–	–	–	–	–	–	–	–	–	–	–	–	–	G	–	–	–	–	–	–	C	C	C	1
16HB36	A	G	–	–	–	–	–	–	–	A	–	–	–	–	–	–	–	–	–	–	–	–	–	–	–	–	–	–	–	–	A	–	–	–	–	–	–	–	1
16HB37	–	–	–	–	–	–	–	–	–	–	–	–	–	–	–	–	–	–	T	–	–	–	–	C	–	–	–	–	–	–	–	–	–	–	–	–	–	–	1
16HB38	–	–	–	–	–	–	–	–	–	–	–	–	–	–	–	–	–	–	T	G	–	–	–	–	–	T	–	–	–	–	–	–	–	–	–	–	–	–	1
16HB39	A	–	–	–	–	–	–	–	–	–	–	–	–	–	–	–	–	–	–	G	–	–	–	–	–	–	–	C	–	–	–	–	–	–	–	–	–	–	1
16HB40	–	–	C	–	–	–	–	–	–	–	–	–	–	–	–	–	–	–	–	G	–	–	–	–	–	–	–	–	–	–	–	–	–	–	–	–	–	–	1
16HB41	–	–	–	–	–	–	–	–	–	G	–	–	–	C	–	–	–	–	–	–	–	–	–	–	–	–	–	–	G	–	–	–	–	–	–	–	–	C	1
16HB42	–	–	–	–	–	–	–	–	–	–	G	–	–	–	–	–	–	–	–	–	–	–	–	–	–	–	–	–	–	–	–	–	–	–	–	–	–	–	1
16HB43	–	–	–	–	–	–	–	–	–	G	–	–	–	–	–	–	–	–	–	–	–	–	–	–	–	–	–	–	G	–	–	–	–	–	T	–	C	C	1
16HB44	–	–	–	–	–	–	–	–	A	–	–	–	–	–	–	–	–	–	–	–	–	–	–	–	–	–	–	–	–	–	–	–	C	–	–	–	–	–	1
16HB45	–	–	–	–	–	T	–	–	–	G	–	–	–	–	–	–	–	–	–	–	–	–	–	–	–	–	–	–	G	–	–	–	–	–	–	–	–	C	1
Reference AA	K	R	F	R	L	T	L	H	D	D	I	L	E	L	A	N	C	K	H	L	P	D	K	E	R	Q	M	N	N	E	E	T	L	V	R	G	C	S	
AA Position	4	5	9	17	19	24	26	31	32	32	34	35	36	45	53	65	70	75	85	90	102	105	115	120	136	157	12	29	29	34	35	64	67	74	77	88	94	95	
AA Variant	–	G	–	–	V	I	M	Y	N	E	M	V	Q	P	–	S	–	T	Y	V	L	Y	T	D	K	H	I	H	S	–	–	–	–	L	C	R	–	–	
Secondary structure	–	–	–	–	–	–	–	–	S	S	S	S	H	–	H	–	–	–	S	S	–	S	–	–	S	S	H	–	–	H	H	–	–	–	S	–	–	–	

The nucleotides matching the reference (GenBank: K02718) are marked with a dash (–), AA, amino acid; S, strand; H, helix

### Phylogenetic analysis of *E6* and *E7*

**Fig. 1:**
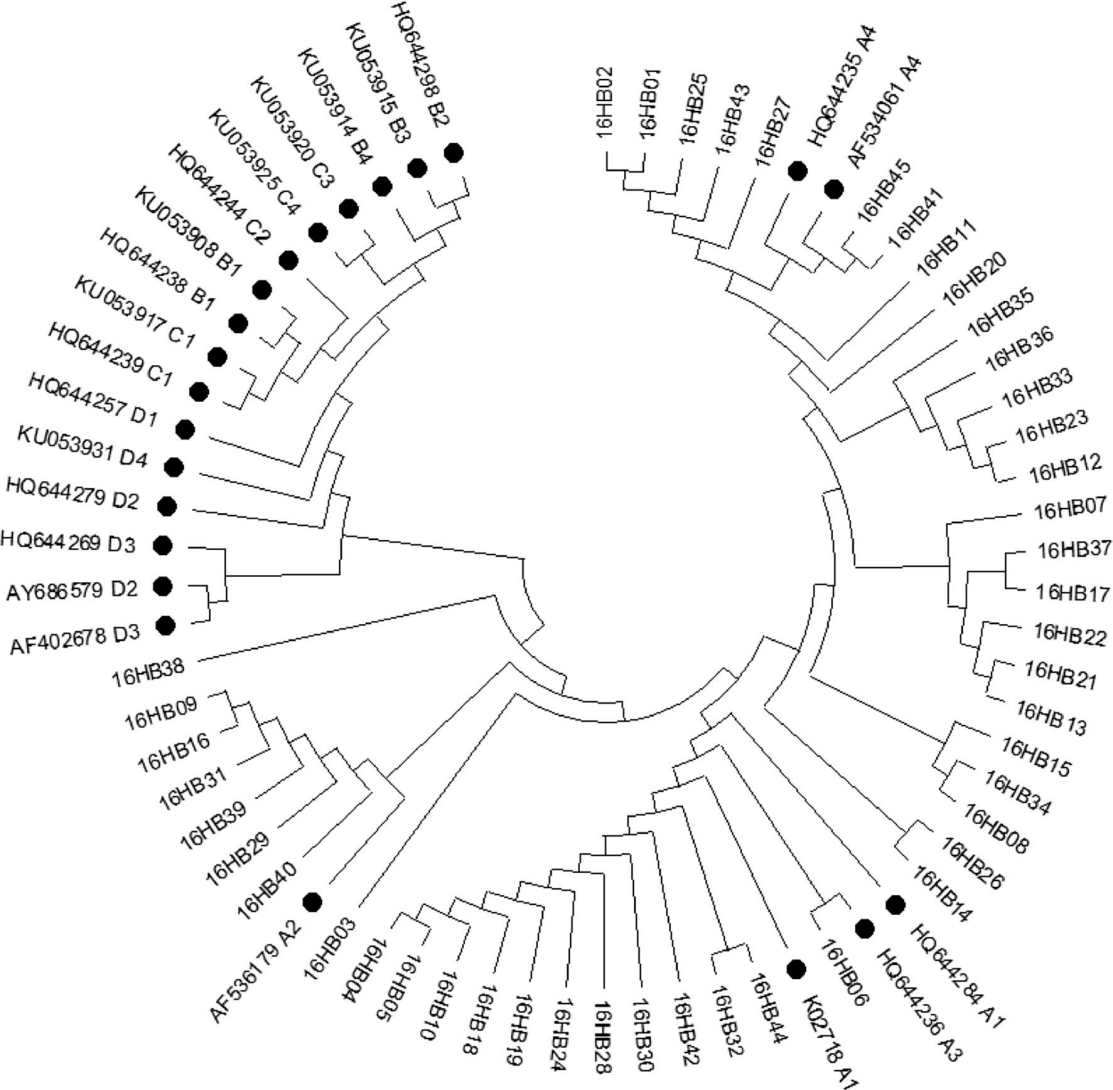
Maximum Likelihood phylogenetic tree of HPV‐16 *E6*. A1–4, B1–4, C1–4 and D1–4 represented the reference sequences of sub-lineages, and the others were variant sequences

**Fig. 2:**
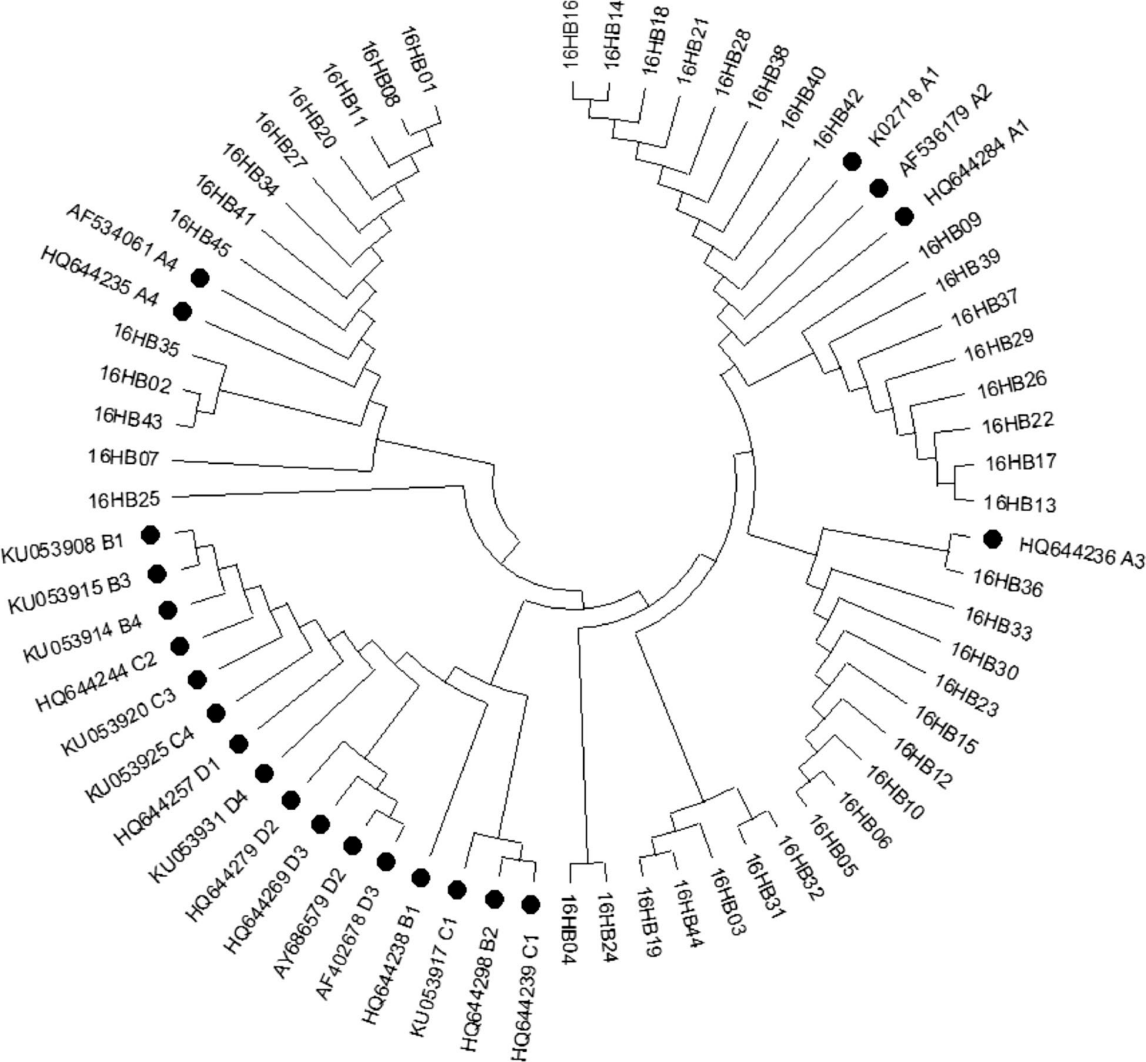
Maximum Likelihood phylogenetic tree of HPV‐16 *E7*. A1–4, B1–4, C1–4 and D1–4 represented the reference sequences of sub-lineages, and the others were variant sequences

Phylogenetic analysis was performed by comparing *E6 and E7* nucleotide sequences, including 45 viral variant sequences and 16 viral reference sequences mentioned above. The phylogenetic tree of *E6* sequence of HPV-16 was shown in Fig. 1. According to the phylogenetic tree of *E6* sequence of HPV-16, all variants were distributed in lineage A, with the most in sub-lineage A4 (119/168, 70.8%), followed by A1 (24/168, 14.3%), A2 (20/168, 11.9%) and A3 (5/168, 3.0%). The phylogenetic tree of *E7* sequence of HPV-16 was shown in Fig. 2. According to the phylogenetic tree of *E7* sequence of HPV-16, all variants were distributed in lineage A, with the most in sub-lineage A4 (102/168, 60.7%), followed by A3 (43/168, 25.6%) and A1 (23/168, 13.7%).

### Selective pressure analysis

No positively selected sites were found in *E6* and *E7* sequences, and the results were shown in Tables 3 and 4.

**Table 3:** Results of PAML estimation for HPV-16 *E6*–*E7* genes

Gene	Model	In likelihood	Parameter estimates
*E6*–*E7*	M0 (one-ratio)	− 1456.339336	ω = 0.53573
	M1 (neutral)	− 1450.807799	p_0_ = 0.63531 p_1_ = 0.36469
	M2 (selection)	− 1448.843792	p_0_ = 0.77049 p_1_ = 0.00000 p_2_ = 0.22951 ω_2_ = 2.34961
	M3 (discrete)	− 1449.747371	p_0_ = 0.64437 p_1_ = 0.12615 p_2_ = 0.22948 ω_0_ = 0.00000 ω_1_ = 0.00000 ω_2_ = 2.35011
	M5 (gamma)	− 1449.081486	α = 0.16628 β = 0.26303
	M7 (beta)	− 1450.874346	p = 0.00557 q = 0.00842
	M8 (beta and ω)	− 1448.843791	p_0_ = 0.77049 p = 0.00500 q = 52.87609 (p_1_ = 0.22951) ω = 2.34960

**Table 4:** Results of likelihood ratio tests of positive selection

Gene	Model 1	Model 2	LRT	*P* value	d_N_/d_S_ > 1 detected?	Positively selected codons
*E6–E7*	M1	M2	3.928014	0.140296113	Yes	None
	M1	M3	2.120856	0.547716257	Yes	None
	M7	M8	4.061110	0.131263306	Yes	None

### B-cell epitope prediction

B-cell epitope prediction was based on the corresponding amino acid reference sequences and variant sequences of HPV-16 *E6* and *E7*. Only epitope prediction scores greater than 0.85 were listed, and the predicted results were shown in Table 5. For the E6 protein, there were10 B-cell epitopes in the variant sequence, of which 6 B-cell epitopes were novel (30–45 IHEIILECVYCKQQLP, 61–76 YRDGNPYAVCDKCLTF, 57–72 LCIVYRDGSPYAVCDK, 67–82 YAVCDKCLTFYSKISE, 27–42 QTTIYDIILECVYCKQ, and 10–25 QDPQERPRKVPQLCTE) and 4 B-cell epitopes also existed in the reference sequence. According to the score ranking, the top four B-cell epitopes predicted by E6 protein were all novel epitopes, namely 30–45 IHEIILECVYCKQQLP (0.91), 61–76 YRDGNPYAVCDKCLTF (0.88), 57–72 LCIVYRDGSPYAVCDK (0.87), and 67–82 YAVCDKCLTFYSKISE (0.87). For the E7 protein, the reference and variant sequences shared a common epitope 39–54 DGPAGQAEPDRAHYNI (0.85).

**Table 5:** Predicted B‐cell epitopes of the HPV‐16 *E6* gene

Reference sequence		Start position	Score	Variants sequence		Start position	Score
Rank	Sequence			Rank	Sequence		
1	YSLYGTTLEQQYNKPL	88	0.87	1	IHEIILECVYCKQQLP	30	0.91
2	RDLCIVYRDGNPYAVC	55	0.86	2	YRDGNPYAVCDKCLTF	61	0.88
2	FHNIRGRWTGRCMSCC	132	0.86	3	LCIVYRDGSPYAVCDK	57	0.87
3	TAMFQDPQERPRKLPQ	6	0.85	3	YAVCDKCLTFYSKISE	67	0.87
				4	YSVYGTTLEQQYNKPL	88	0.86
				4	QTTIYDIILECVYCKQ	27	0.86
				4	RDLCIVYRDGNPYAVC	55	0.86
				4	FHNIRGRWTGRCMSCC	132	0.86
				5	TAMFQDPQERPRKLPQ	6	0.85
				5	QDPQERPRKVPQLCTE	10	0.85

## Discussion

Cervical cancer is the leading cause of cancer deaths among women in developing countries [20]. Globally, the most common HR-HPV types in cervical cancer are HPV-16 (15.56–83.78%) and HPV-18 (3.4–41.1%) [21]. HPV-16 is the major cause of more than half of cervical cancer worldwide and the main HPV type causing invasive cervical cancer. There are significant geographic and ethnic differences in HR-HPV infection types [22, 23]. In China, HPV-52 and HPV-58 are the most common types, followed by HPV-16, which differed from the HPV type spectrum in other countries [24–26]. In Jingzhou, central China, the three major HR-HPV types in women are HPV-52, HPV-58, and HPV-16, respectively [27].

The E6 and E7 proteins encoded by the *E6* and *E7* genes in HPV-16 are major oncogenic proteins and play important roles in viral replication, termination of cell differentiation and oncogenesis [28]. Previous studies have shown that HPV variants may affect viral infectivity, pathogenicity and host immune response [29]. Therefore, it is meaningful to analyze *E6* and *E7* gene variation and corresponding amino acid sequences. The sequencing results of *E6* and *E7* sequences indicated that the variation rate of *E6* sequences (5.5%) was greater than that of *E7* sequences (4.0%), implying *E7* sequences were more conserved than *E6* and more suitable as a target for therapeutic vaccine development. In this study, T178G (D32E) (102/168, 60.7%) of *E6* and A647G (N29S) (101/168, 60.1%) and T846C (102/168, 60.7%) of *E7* were the dominate variants, which were also found in other regions of the world. In Korea, the most prevalent variants are *E6* T178G (68%) and *E7* A647G (73%) in HPV-16 [30]. In India, the most frequent variants are T350G (100%) in *E6* and T789C (87.5%) in *E7* [31]. In Xinjiang, China, the most frequent variants in *E6* are T350G (36/75, 48%) and T178G (19/75, 25.3%); the most frequent variant in *E7* is A647G (18/ 75, 24%) [32]. In Northeast China, the most common variants are T178G (32.69%) in *E6* and A647G (34.62%), G666A (38.46%) and T846C (32.69%) in *E7* [33]. 16HB01, which simultaneously had the variants T178G (D32E), A647G (N29S) and T846C, was the most prevalent (58/168, 34.5%) in the 45 variant groups. In Taizhou, China, T178G (D32E) in *E6* and A647G (N29S) and T846C in *E7* occur in 96.4% of the A4 (Asian) variants [34]. In this study, twelve nonsynonymous variants in the sequences encoding E6 or E7 proteins were found, which may affect the folding of oncoproteins in the secondary structure and result in differences in their ability to interact with tumor suppressor proteins and the pathogenicity of HPV-16. T292C, G597A, G753C and G781 were new variants that had never been reported before. Specific mutations of HPV-16 are associated with an increased risk of high-grade squamous intraepithelial lesions and invasive cervical cancer development [35]. In Swedish, prototype HPV-16 and its *E6* variant L83V are both prevalent in preinvasive and invasive cervical lesions [36]. In Shanghai, China, T7220G (D32E) variation in *E6* and A7689G (N29S) in *E7* increase the incidence of HSIL [37]. In Kunming, China, the C749T (S63F) variation in *E7* is associated with cervical cancer [9]. In this study, A647G (N29S) variation in *E7* was significantly related to the higher risk of HSIL and cervical cancer, though the sample size of cervical cancer is relatively limited due to the fact that the patients in this study were mainly outpatients underwent HR-HPV screening.

HPV-16 is divided into genetic sub-lineages A1–4, B1–4, C1–4 and D1–4. The distribution of the lineages varies geographically and ethnically [38]. Globally, variants of HPV-16 sub-lineages A1–A3 are predominantly found in Europe, A4 sub-lineage in Asia, B and C lineages exclusively in Africa, and D lineage most common in South/Central America [39]. Different lineages exhibit disparity in carcinogenic potential. In comparison to other lineages, A4 sub-lineage and D lineage often show more strong association with cervical cancer [39, 40]. The worldwide burden of cervical cancer in different HPV lineages is largely driven by the spread of the historical HPV-16 sub-lineages, and HPV-16 gene variants can significantly affect the risk of cervical cancer development [39, 41]. In this study, we obtained 176 complete *E6* and *E7* gene sequences from HPV-16 isolates. All HPV-16 variants belonged to the A lineage, with the A4 sub-lineage predominating (70.8% in E6 and 60.7% in E7). Our findings were consistent with the data from North China (64.70% of A4) and East China (62.50% of A4) [40, 42].

The main feature of positive selection is that it incurs an abnormal increase in allele frequency, which allows the virus to adapt rapidly to environmental changes [43]. In Greek population, the selection pressure analysis of amino acid residues of HPV-16 shows that codon 83 of E6 protein and codon 85 of E7 protein are positive selection sites [44]. However, in this study, positively selected site of HPV-16 *E6* and *E7* sequences was never found in Jingzhou, central China, which was also in consistence with the results in southwest China [45]. This may be one of the possible reasons why HPV-16 is not the most prevalent in China, though needed to further verify in the future.

The HPV vaccines currently available on the market are preventive vaccines, and the development of therapeutic vaccines for people who have been infected with HPV is of great application prospects. E6 and E7 proteins are persistently expressed in HPV infection cells, most cervical cancer and precancerous lesions, but not in normal tissues [46]. Therefore, HPV E6 and E7 proteins are ideal targets for diagnostic detection and therapeutic vaccine design. In this study, the B-cell epitopes of HPV-16 E6 and E7 proteins were predicted using the ABCpred server, and the effects of amino acid changes caused by genetic variants on B-cell epitopes were analyzed based on changes in epitope fraction. The results predicted 10 B-cell epitopes in E6 protein, of which six were new epitopes never encoded by the reference sequence. In addition, non-conservative substitutions of some amino acids improved B-cell epitope prediction scores, such as H31Y, D32N, D32E, I34M, L35V, E36Q, L45P, N65S, and K75T. Therefore, non-conservative substitutions of amino acids should be fully considered when developing therapeutic vaccines. The reference sequence and variant sequence of *E7* predicted the B-cell epitope at the same site (39–54 DGPAGQAEPDRAHYNI). The optimal epitope for therapeutic vaccines is often selected from the same region of the reference sequence and the variant sequence [47]. Therefore, E7 protein may be more suitable as an ideal target for therapeutic vaccine design. These results are valuable for the development of HPV-16 therapeutic vaccines for the population in specific region, though these predicted epitopes still need to be further verified in *vivo*.

## Conclusion

This study researched on the genetic variability of HPV-16 *E6* and *E7* genes in Jingzhou, central China. The sequencing results showed *E7* sequences were more conservative than *E6* sequences, meanwhile several novel variations (T292C, G597A, G753C and G781C) were detected. All HPV-16 variants belonged to the A lineage, with the A4 sub-lineage predominating. In addition, non-conservative substitutions, H31Y, D32N, D32E, I34M, L35V, E36Q, L45P, N65S and K75T in *E6*, affected multiple B-cell epitopes. This study provided useful data for the epidemiological characteristics, immunotherapy and vaccine development of HPV-16, in central China.

## Data Availability

The data generated during the current study are available in the NCBI repository (Home—Nucleotide—NCBI (nih.gov)). The sequence data were submitted to GenBank with accession numbers OQ659416-OQ659460.

## Abbreviations

HPV-16: Human papillomavirus type 16
HR-HPV: High-risk human papillomavirus
PCR: Polymerase chain reaction
LCR: Long control region
LSIL: Low-grade squamous intraepithelial lesion
HSIL: High-grade squamous intraepithelial lesion
The amino acid acronyms H, Y, D, N, E, I, M, L, V, Q, P, S, K and T: Histidine, tyrosine, aspartic acid, asparagine, glutamic acid, isoleucine, methionine, leucine, valine, glutamine, proline, serine, lysine and threonine
